# Wearable multimode sensor with a seamless integrated structure for recognition of different joint motion states with the assistance of a deep learning algorithm

**DOI:** 10.1038/s41378-022-00358-2

**Published:** 2022-02-17

**Authors:** Lei Wen, Meng Nie, Pengfan Chen, Yu-na Zhao, Jingcheng Shen, Chongqing Wang, Yuwei Xiong, Kuibo Yin, Litao Sun

**Affiliations:** SEU-FEI Nano-Pico Center, Key Laboratory of MEMS of Ministry of Education, School of Electronic Science & Engineering, Southeast University, Nanjing, 210096 P. R. China

**Keywords:** Electrical and electronic engineering, Electronic properties and materials, Structural properties, Structural properties

## Abstract

Accurate motion feature extraction and recognition provide critical information for many scientific problems. Herein, a new paradigm for a wearable seamless multimode sensor with the ability to decouple pressure and strain stimuli and recognize the different joint motion states is reported. This wearable sensor is integrated into a unique seamless structure consisting of two main parts (a resistive component and a capacitive component) to decouple the different stimuli by an independent resistance-capacitance sensing mechanism. The sensor exhibits both high strain sensitivity (GF = 7.62, 0–140% strain) under the resistance mechanism and high linear pressure sensitivity (*S* = 3.4 kPa^−1^, 0–14 kPa) under the capacitive mechanism. The sensor can differentiate the motion characteristics of the positions and states of different joints with precise recognition (97.13%) with the assistance of machine learning algorithms. The unique integrated seamless structure is achieved by developing a layer-by-layer casting process that is suitable for large-scale manufacturing. The proposed wearable seamless multimode sensor and the convenient process are expected to contribute significantly to developing essential components in various emerging research fields, including soft robotics, electronic skin, health care, and innovative sports systems applications.

## Introduction

Monitoring and recognizing motion features of the human body with wearable sensors are essential functions for understanding human activities and vital signs, especially in applications such as intelligent medical rehabilitation, smart sport exercise, soft robotics, and electronic skin^1–6^. However, human motion is a complex process involving multiple joints, and each joint motion consists of both the bending/compressing of the skeleton and the stretching of the skin covered in the surface of the bone. To date, typical flexible sensors usually contact the surface of human bodies; thus, these sensors are stimulated by both compressive pressure and stretching strain. Under different stimuli, the majority of sensors yield similar electrical output, making it challenging to distinguish these mechanical inputs^7–9^. Because the bending/compression and stretching of each joint occur together, it is highly necessary to develop a multimode wearable sensor to monitor and decouple the multiple parameters of the joint’s motions^10,11^.

In recent years, three strategies have been proposed to design multimode flexible sensors. The first is to design a single structure sensor to monitor different stimuli simultaneously^12–15^. Song et al. deposited multiwall carbon nanotubes (MWCNTs) onto a wrinkled fabric surface to measure tangential stretching forces and normal pressure with opposite resistance responses^16^. Qin et al. proposed a multifunctional epidermal sensor by immersing hydrophobic carbon nanotubes (CNTs) into a polyacrylamide hydrogel, which exhibited high tensile strain sensitivity in wide strain ranges and high linear sensitivity in a large pressure region^17^. Chao et al. presented a multifunctional skin-like sensor through a dopamine-triggered gelation route. The sensor was sensitive to strain, pressure, and temperature^18^. However, it remains difficult for a single-structure strategy to distinguish different stimuli. The second strategy is to design two discrete sensors and assemble them together in a certain way to detect different stimuli^19,20^. Su et al. applied NaCl-doped agarose gel as a biocompatible conductive filler and injected it into a 3D printed elastomer shaper for wearable sensors. The sensors exhibited the ability to differentiate bending and stretching motions by assembling both straight and spring channels inside the elastomer shaper^21^. Lee et al. fabricated multidirectional strain sensors by cross-compacting two anisotropic carbon nanofiber sensors together, which realized stretching strains parallel and perpendicular to sensor alignment^22^. These assembled sensors solved the decoupling problem of multiple stimuli well. However, the mechanical properties and stability are always challenging, and the manufacturing process is relatively complicated. The third is to design a single structure as the sensor with two integrated independent parts^23^. Park et al. presented stretchable energy harvesting e-skin, which consisted of PDMS/SWNT-film/porous PDMS/PDMS spacers/SWNT-film/PDMS (from top to bottom). The sensor can detect and differentiate pressure and strain signals simultaneously^24^. However, the complicated process flow increased both the difficulty of fabrication and the cost of the sensor. It is critical to develop a wearable multimode sensor with convenient manufacturing to decouple multiple parameters.

In this study, we present a wearable seamless resistance-capacitance structural multimode (SRCSM) sensor that can decouple the pressure and stretchable strain applied on each joint during human motion. The SRCSM sensor is integrated into a unique seamless structure, which consists of two different main parts (a resistive component and a capacitive component) to decouple the different stimuli by an independent resistance-capacitance sensing mechanism. A layer-by-layer casting process is proposed, which is convenient and suitable for large volume manufacturing. Furthermore, the SRCSM sensor can differentiate the motion characteristics of the positions and states of different joints with precise recognition (97.13%) with the assistance of machine learning algorithms. These demonstrations indicate that the proposed SRCSM sensor and processing technique have high potential value in various emerging research fields, including soft robotics, health care monitoring, and intelligent sports systems applications.

## Results and discussion

### Fabrication of the SRCSM sensor

**Fig. 1:**
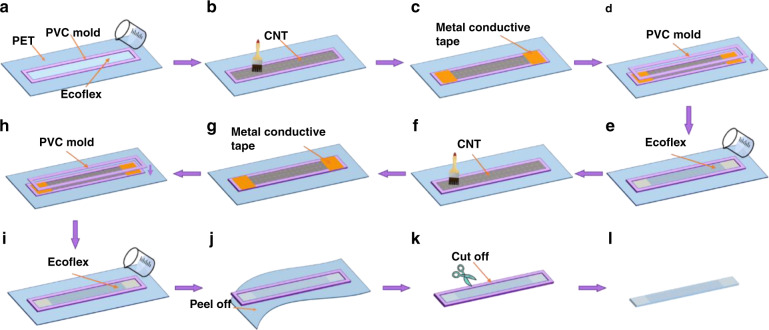
Fabrication process of the SRCSM sensor. **a** Casting Ecoflex with PVC mold. **b** Brushing MWCNTs on the semicured Ecoflex. **c** Gluing on the conductive metal tape. **d**–**g** Repeating steps **a**–**c**. **i** Casting Ecoflex as an encapsulated layer with PVC mold. **j** Curing ecoflex and peeling off from the PET substrate. **k**–**l** Cutting off the PVC mold and obtaining the SRCSM sensor

The layer-by-layer casting processable fabrication of the SRCSM sensor is schematically illustrated in Fig. 1. First, a polyvinyl chloride (PVC) mold (600 µm in depth, fabricated by laser cutting) was attached to a polyethylene terephthalate (PET) substrate. Parts A and B of Ecoflex were mixed at a weight ratio of 1:1 and then poured into the PVC mold after the bubbles were dissipated. The solutions were cured for 50 min to obtain the semicured Ecoflex (Fig. 1a). Second, the MWCNTs (1 mg/cm^2^) were pressed into the surface of semicured Ecoflex by brushing. Due to the pressure during brushing, part of the MWCNTs were embedded into the incompletely cured Ecoflex. (Fig. 1b). After 3 hours of full curing, a hybrid MWCNT/Ecoflex thin film layer formed on the surface of Ecoflex with some MWCNTs embedded into the surface of Ecoflex. Conductive metal tapes were attached to the hybrid MWCNT/Ecoflex thin film layer as the lead electrodes (Fig. 1c). Then, the above steps were repeated twice, as shown in Fig. 1d to g. Subsequently, the casting process of Ecoflex was repeated to encapsulate the structure (Fig. 1i). Ecoflex was fully cured for 3 hours and peeled off from the PET substrate, as illustrated in Fig. 1j. Finally, the PVC mold was cut off, and the SRCSM sensor was obtained, as shown in Fig. 1k, l. All fabrication steps were completed at room temperature (20 °C, 50% humidity).

### Morphology characterization of the SRCSM sensor

**Fig. 2:**
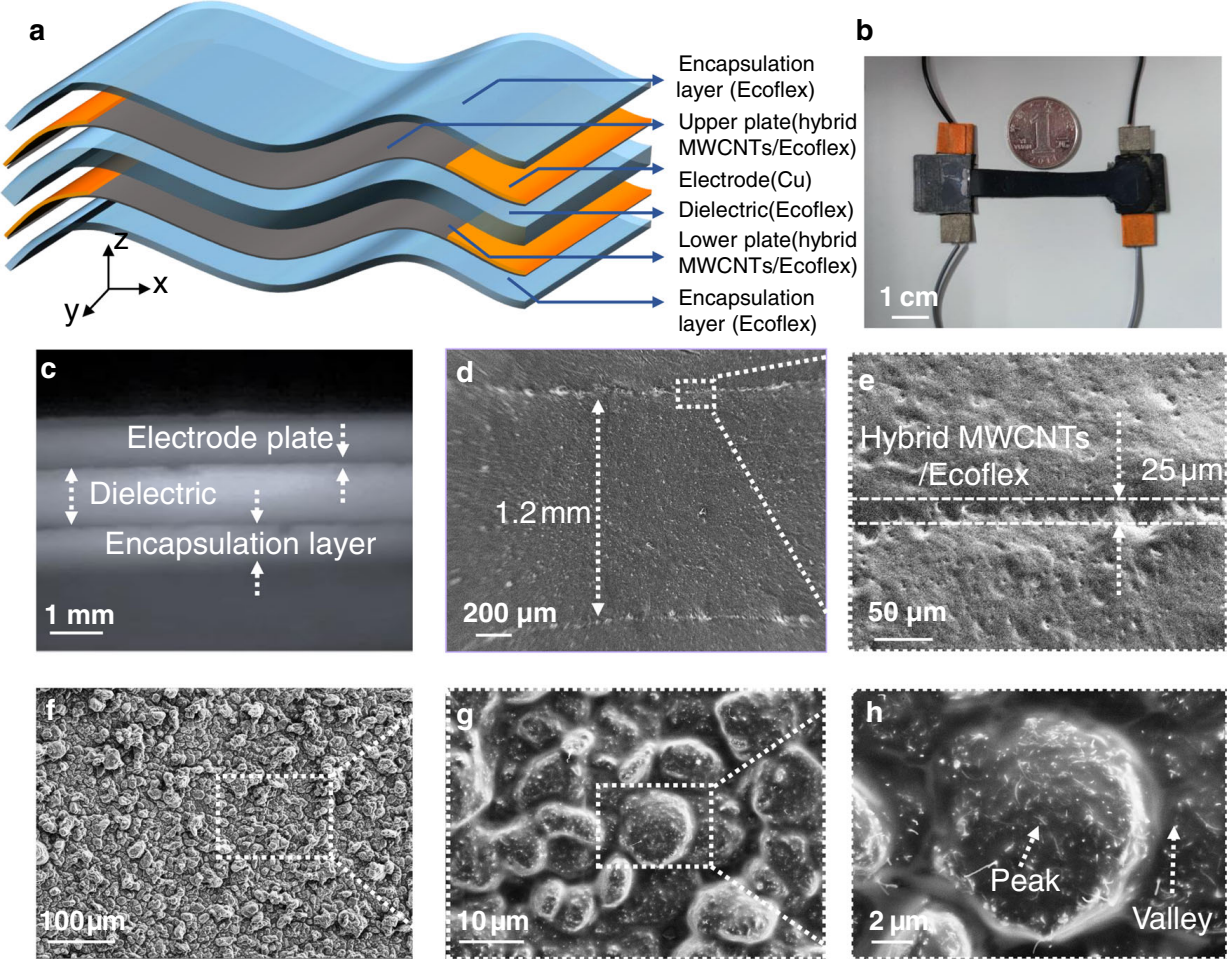
Morphology characterization of the SRCSM sensor **a** Exploded view of the SRCSM sensor. **b** Photograph of the integrated SRCSM sensor. **c**–**e** Cross-sectional photograph and SEM images of the SRCSM sensor. **f**–**h** SEM surface topography of the SRCSM sensor

Figure 2 shows the conceptual structural design and morphology characterization for the proposed SRCSM sensor. As shown in Fig. 2a, the two facesheets of the sandwich-shaped sensor made of hybrid Ecoflex/MWCNT films served as the electrodes, and pure Ecoflex served as the dielectric layer for the capacitive component. Copper tapes were attached to the facesheets for measuring output signals. Two Ecoflex layers were used to encapsulate the sensor. Additionally, the facesheet of the electrode was utilized as an independent resistive component. Figure 2b shows the appearance of the SRCSM sensor. Figure 2c–e shows the cross-sectional photography and SEM images of the proposed sensor. The thickness of each electrode plate is approximately 25 µm, the dielectric layer is approximately 1.2 mm, and the encapsulation layer is 600 µm. Figure 2f–h displays the microsurface topography of the electrodes, which is similar to our previous research^25^. A brush was used to press MWCNTs into the semicured Ecoflex surface. Under the action of force, the MWCNTs were embedded in the Ecoflex surface and formed a hybrid conductive layer, which was used as the electrode. Figure 2f–h also shows that MWCNTs are evenly distributed on Ecoflex. Furthermore, due to the discrete nature of the bristles, a microstructure with peaks and valleys was formed on the surface of the hybrid layer during the brushing process. Thus, the resistive component can be regarded as a double-layer structure: one is the peak-valley microstructured hybrid layer, and the other is the flat hybrid layer at the bottom of the valley. The length and width directions of the sensor are denoted as the x-axis and y-axis, and the through-thickness direction is represented as the z-axis. The resistance (*R*) and capacitance (*C*) values of the SRCSM sensor can be simultaneously changed under mechanical stimuli such as in-plane stretching strain (*ε*) in the x-axis or through-thickness compressive pressure (*p*) in the z-axis.

### Mechanical, electrical property and sensing mechanism of the SRCSM sensor

**Fig. 3:**
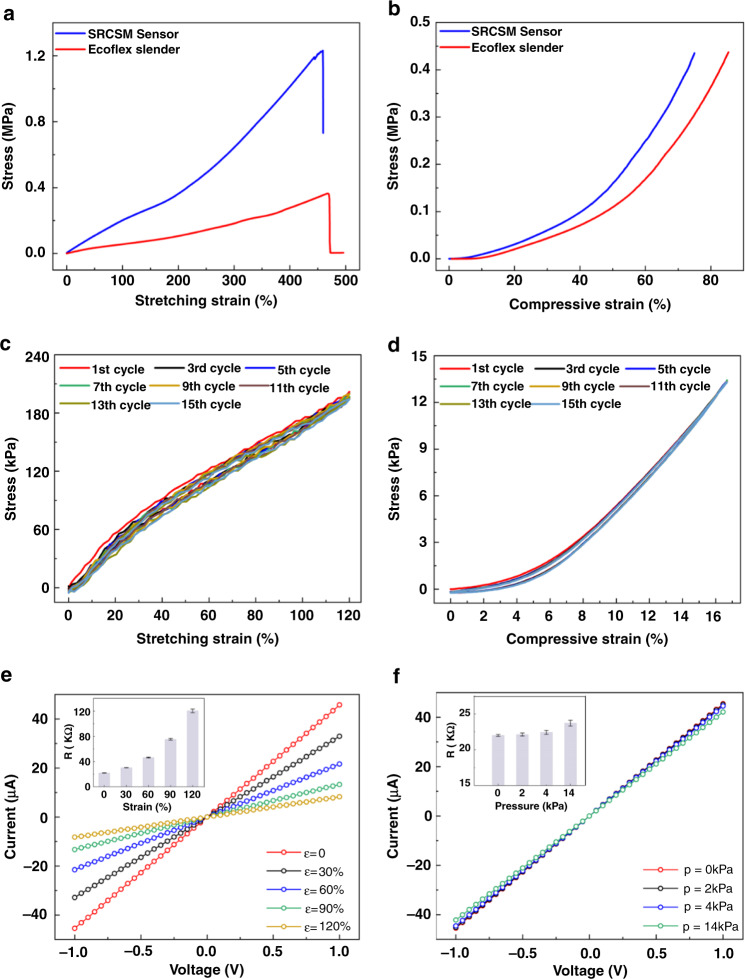
Mechanical and electrical properties of the SRCSM sensor. **a** Stretching stress–strain curves of the SRCSM sensor and the pure slender Ecoflex. **b** The compressive stress–strain curves of the SRCSM sensor and pure slender Ecoflex. **c** Fifteen successive cyclic loading–unloading curves of the SRCSM sensor at a maximum stretching strain of 120%. **d** Fifteen successive cyclic loading–unloading curves of the SRCSM sensor at 17.5% strain (ca. 14 kPa, compressive strength). **e** The curves under various stretching stains. **f** The curves under various pressures. The applied voltage is from −1 V to 1 V. (Insets: The average resistance (*R*) under different applied stimuli)

To quantitatively evaluate the mechanical properties, stretching and compressive tests were investigated. Figure 3a illustrates the stretching stress–strain curves of the SRCSM sensor and the pure Ecoflex slender (the stretching rate was 10.1 mm/min). The Young’s modulus of the seamless structure sensor is 269 kPa, and the fracture strain is 458%. The identical tests were given to the neat Ecoflex slender with the same geometries to make comparisons. The Young’s modulus of the pure Ecoflex slender is 77 kPa, and the fracture strain is 469%. Introducing hybrid Ecoflex/MWCNT electrode layers has a specific toughening effect on the seamless structure and has little effect on the fracture strain. Similarly, the mechanical compressive properties of the SRCSM sensor were also evaluated by compressive models (the compressive rate was 0.5 mm/min). The compressive stress–strain curves of the SRCSM sensor are shown in Fig. 3b. Representative stretching loading–unloading tests at 120% strain are displayed in Fig. 3c. All hysteresis loops remained almost unchanged during 15 cycles, indicating the excellent fatigue resistance of the SRCSM sensor due to the elastic characteristics. In addition, cyclic compressive tests at 17.5% strain (ca. 14 kPa, compressive strength) revealed the excellent elasticity of the SRCSM sensor (Fig. 3d). Such stable and tough mechanical characteristics of the SRCSM sensor are realized due to the overall seamless structure rather than two independent sensing devices assembled.

The direct current-voltage characteristic curves of the SRCSM sensor under different applied stretching strains and pressures are also shown in Fig. 3e and f. The currents were recorded in the voltage range of −1 to 1 V. The high linear behaviors of the curves demonstrate that the device has an excellent ohmic contact, and the reciprocal slope of the *I − V* curves can be used to estimate the resistance of the sensor. As shown in the insets in Fig. 3e, the average resistances under different stretching strains of 0%, 30%, 60%, 90%, and 120% are 22 kΩ, 30.4 kΩ, 46.5 kΩ, 75.3 kΩ, and 120.6 kΩ, respectively. This indicates that the resistance of the electrode increases rapidly with the applied strain. Similarly, the inset in Fig. 3f illustrates that the resistances are 22 kΩ, 22.1 kΩ, 22.4 kΩ and 23.7 kΩ under 0 kPa, 2 kPa, 4 kPa, and 14 kPa, respectively. Compared with stretching stimuli, the resistance of the sensor under different pressure stimuli is relatively stable. The bias-free resistance will simplify the relationship between the output electrical signals and the applied strains and the data process integrated circuit.

**Fig. 4:**
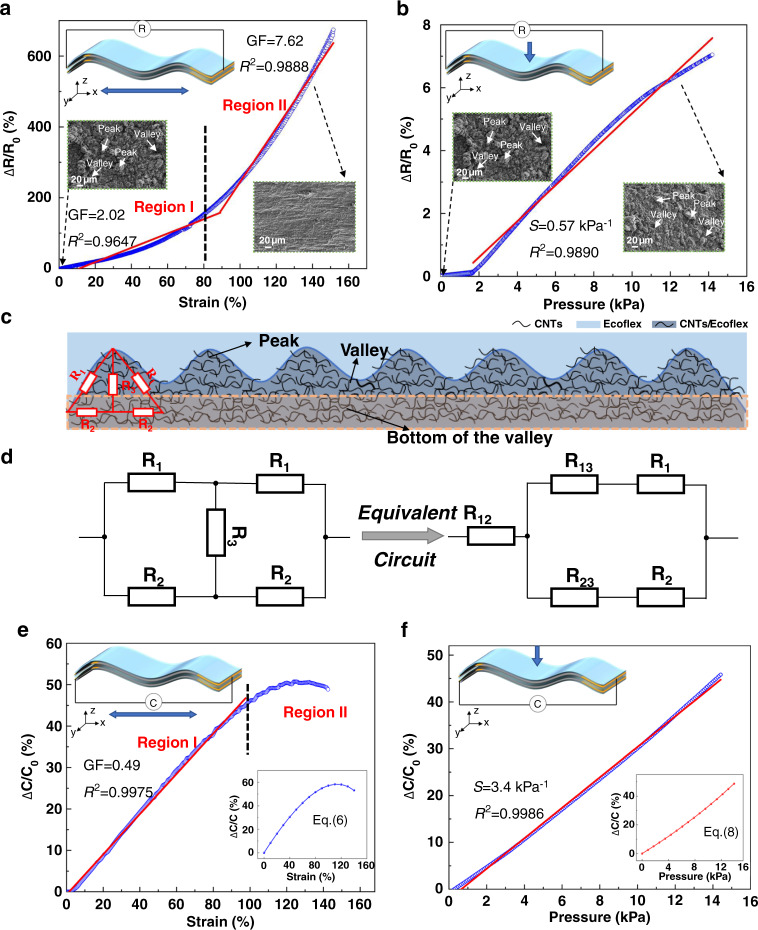
Sensing performance of the SRCSM sensor. **a**, **b** Relative resistance variation ratio (Δ*R*/*R*_0_) as a function of the applied stretching strain and pressure. The insets are SEM surface topographies of the SRCSM sensor under the different stimuli. **c** The electrical modeling of the SRCSM sensor. **d** The transformation of the Δ − Y equivalent circuit based on Thevenin’s theorem. **e**, **f** Relative capacitance variation ratio (Δ*C*/*C*_0_) as a function of the applied stretching strain and pressure. The blue dots are experiments results. The solid red lines are the corresponding linear fitting lines, and the fitting coefficients are also indicated. The insets: Theoretical curves

The gauge factor of the sensor is an important parameter to evaluate the sensing performance of the device. It is usually defined as GF = (Δ*R*/*R*_0_*)*/*ε*, where *R*_0_ is the initial resistance, Δ*R* denotes the change in the resistance before and after the application of strain, and *ε* represents the applied strain. Figure 4a indicates the typical relationship between the relative resistance variation ratio (Δ*R*/R_0_) and the applied stretching strain *ε* (up to 140%) of the sensor at a stretching rate of 10.1 mm/min. The characteristic curve can be divided into two distinct regions with GF values of 2.02 (0 < ε < 80%) and 7.62 (80–140%), which depend on the peak-valley synergetic conductive paths in the resistive structure. To further reveal the sensing mechanism from electrical modeling, the resistance *R* was decomposed into three parts: *R*_1_, *R*_2_, and *R*_3_, representing the resistance of the slope of the peak, the bottom of the valley and the interior of the peak, respectively. The detailed electrical model is shown in Fig. 4c. To simplify the circuit and facilitate the calculation, the circuit is equivalent based on Thevenin’s theorem, as shown in Fig. 4d. The overall equivalent circuit resistance is described as:

1 $$R={R}_{12}+\frac{({R}_{13}+{R}_{1})\ast ({R}_{23}+{R}_{2})}{{R}_{13}+{R}_{1}+{R}_{23}+{R}_{2}}$$

2 $${R}_{12}=\frac{{R}_{1}\ast {R}_{2}}{{R}_{1}+{R}_{2}+{R}_{3}},\,{R}_{13}=\frac{{R}_{1}\ast {R}_{3}}{{R}_{1}+{R}_{2}+{R}_{3}},\,{R}_{23}=\frac{{R}_{2}\ast {R}_{3}}{{R}_{1}+{R}_{2}+{R}_{3}}$$

In region I (0 < ε < 80%), the bottom of the valley was stretched first, causing a large number of conductive paths to be destroyed, and *R*_2_ increased sharply and almost infinitely. However, the slope of the peak becomes slow under stretching strain, and the interior of the peak is squeezed, resulting in a slight decrease in *R*_3_ and an almost unchanged *R*_1_. The resistance *R* can be approximated as the series resistance of *R*_12_, *R*_13_ and *R*_1_, where *R*_12_ increases with the increase of *R*_2_, and *R*_13_ decreases with the increase of *R*_2_, making the change of resistance in this region relatively small. As the applied strain gradually increased to region II (80–140%), the peak was gradually stretched, and the peak-valley microstructures almost disappeared, as shown in the insets of Fig. 4a. Therefore, the resistance *R*_1_ increased drastically with increasing strain, and *R*_2_ and *R*_3_ remained almost unchanged. The resistance of *R*_12_ and *R*_13_ both increased with the increase of *R*_1_, making a higher sensitivity in this region.

The sensitivity *S* of the sensor under pressure is defined as *S*=((Δ*R*/*R*_0_*)*/Δ*p*), where Δ*p* represents the value of applied pressure. Figure 4b indicates the typical relationship between the relative resistance variation ratio (Δ*R*/*R*_0_) and the applied pressure (up to 14 kPa) of the sensor at a pressing rate of 0.5 mm/min. The resistance variation ratio (Δ*R*/R_0_) increases with the applied pressure, leading to tangential stretching of the resistive structure. The SRCSM sensor obtains a small *S* of nearly 0.57 kPa^−1^ due to slight tangential stretching. The slow increase in the range of 0–2 kPa is due to the slight decrease in *R*_3_ under pressure, which suppresses the increase in resistance.

Figure 4e indicates the relationship between the capacitance variation ratio Δ*C*/*C*_0_ and the applied stretching strain *ε* (up to 140%) of the sensor at a stretching rate of 10.1 mm/min. For the capacitance strain sensor, GF can be defined as GF = (Δ*C*/*C*_0_)/ε, where *C*_0_ is the initial capacitance and Δ*C* denotes the capacitance change before and after the application of strain. The curve can also be divided into two regions. In region I (0 < ε < 100%), the sensor obtained a linear characteristic curve, and after that, the capacitance variation ratio Δ*C*/*C*_0_ decreased. The electrical model of the sensor is constructed to reveal the working mechanism. The parallel plate capacitance *C* can be defined as *C* ≡ *Q*/*V*, where *Q* is the stored charge and *V* is the electrostatic potential. Ignoring the influence of fringe field effects and according to Gauss’s theorem, the magnitude of electric field *E* is related to charge *Q*, which can be obtained as *E* = *Q*/*ε*_r_ *A*. *ε*_r_ is the relative permittivity, and *A* is the area of the capacitor. Since the voltage value is equal to the product of electric field *E* and electrode plate spacing *d*, the parallel plate capacitance *C* is:

3 $$C\equiv \frac{Q}{V}=\frac{Q}{Ed}=\frac{Q}{\frac{Q}{{\varepsilon }_{r}A}d}=\frac{{\varepsilon }_{r}A}{d}$$

Equation (3) shows that the capacitance *C* has no relationship with the amount of charge *Q* stored in the electrode plate, so the resistance change of the electrode plate can be ignored.

To verify the accuracy of the experimental results under the applied strain, we deduced the formula of the variation ratio of capacitance. The change in the capacitance $\Delta C$ can be defined as Δ*C = C−C*_0_, where *C*_0_ is the initial capacitance and *C* is the capacitance under the applied strain. According to Eq. (1), the capacitance *C* can be expressed as:

4 $$C=\frac{{\varepsilon }_{r}({w}_{0}+\bigtriangleup w)({l}_{0}+\bigtriangleup l)}{{d}_{0}+\bigtriangleup d}$$

where Δ*l*, Δ*w*, and Δ*d* represent the variation of the length, width, and thickness of the device under the applied strain, respectively. According to the calculated formula of Poisson’s ratio (detailed formula derivation can be achieved in the support information in Part 1), the variation ratio of capacitance Δ*C*/*C*_0_ can also be defined as:

5 $$\frac{\bigtriangleup C}{{C}_{0}}=\frac{(1-\frac{\bigtriangleup l}{{l}_{0}}\ast {\nu }_{xy})(1+\frac{\bigtriangleup l}{{l}_{0}})}{1-\frac{\bigtriangleup l}{{l}_{0}}\ast {\nu }_{xz}}-1$$

Δ*l*/*l*_0_ can be written as *ε*, which represents the stretching strain:

6 $$\frac{\bigtriangleup C}{{C}_{0}}=\frac{(1-\varepsilon \ast {\nu }_{xy})(1+\varepsilon )}{1-\varepsilon \ast {\nu }_{xz}}-1$$

where *v*_xy_ and *v*_xz_ are Poisson’s ratios in the *y* and *z* directions, respectively. Poisson’s ratios *v*_xy_ and *v*_xz_ of the structure are 0.45 and 0.3, respectively, which can be calculated by measured data, as shown in Fig. S1. The inset in Fig. 4e shows the functional relationship between the variables Δ*l*/l_0_ and Δ*C*/*C*_0_. In region I (0 < ε < 100%), as the stretching strain increases, the length of the electrode plate increases, the area of the electrode plate increases, and the distance between the electrode plates decreases. Therefore, the capacitance increases with the strain, and the GF value is 0.49. In region II (100% < ε < 140%), as the strain increases to a certain extent, the decrease in width cannot be ignored anymore, resulting in a relatively small increase or even decrease in the area of the electrode plate. The theoretical derivation result shows the same characteristics as the experimental results in Fig. 4e.

The typical relationship between the capacitance variation ratio Δ.*C*/*C*_0_ and the applied pressure of the sensor is shown in Fig. 4f. The sensitivity of the capacitance pressure sensor can be defined as *S* = ((Δ*C*/*C*_0_)/Δ*p*), where Δ*p* represents the value of applied pressure and *C*_0_ represents the original capacitance of the sensor. The *S* value of the capacitance pressure sensor is 3.4 kPa^−1^. As the pressure increases, *d* of the capacitance decreases, *A* of the plates increases, and the capacitance *C* increases sequentially. To verify the linearity of the capacitance variation ratio pressure curves, the equation of Δ.*C*/*C*_0_ under pressure is also derived (the detailed formula derivation can be achieved in support information Part. 2). The variation ratio of capacitance Δ*C*/*C*_0_ is defined as:

7 $$\frac{\bigtriangleup C}{{C}_{0}}=\frac{(1+\frac{\bigtriangleup d}{{d}_{0}}\ast {\nu }_{zx})(1+\frac{\bigtriangleup d}{{d}_{0}}\ast {\nu }_{zy})}{1-\frac{\bigtriangleup d}{{d}_{0}}}-1$$

8 $$\frac{\bigtriangleup C}{{C}_{0}}=\frac{(1+\lambda \ast \bigtriangleup p\ast {\nu }_{zx})(1+\lambda \ast \bigtriangleup p\ast {\nu }_{zy})}{1-\lambda \ast \bigtriangleup p}-1$$

where *λ* represents the relation between the strain Δ*d*/*d*_*0*_ and the applied pressure Δ*p*, and the expression can be obtained by fitting the compressive stress–strain curve of Fig. 3d. Poisson’s ratios *v*_*zx*_ and *v*_*zy*_ of the structure are both nearly 0.66, which can be calculated from Fig. S1. The theoretical derivation result of the capacitance variation ratio-pressure curves is shown in the inset in Fig. 4f, which is consistent with the experimental result.

The sensitivity *S* was converted to the GF value, which made it convenient to obtain comparison results. The GF value of the resistance under pressure is 0.5, which is much lower than the GF under stretching (2.02–7.62). The GF value of the capacitance under pressure is 3.01, which is much higher than the GF under stretching (0.49). It can be revealed that the significant distinction of sensitivity ensures that the proposed sensor possesses excellent decoupling ability. In addition, the layer-by-layer casting process is suitable for large-scale manufacturing. The sensitivity of different batches of samples can be seen in Fig. S2 and Table S1, and the sensitivity of different samples is stable.

**Fig. 5:**
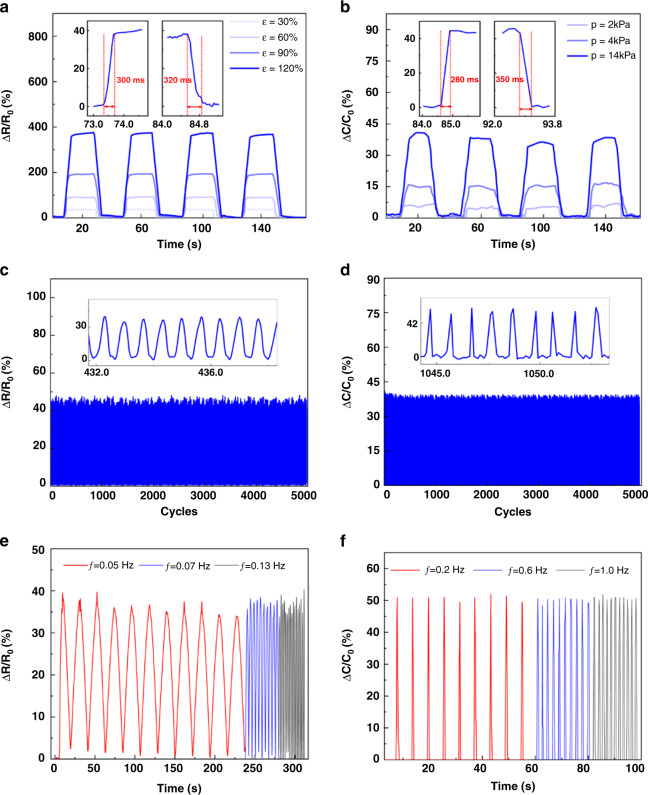
Stability and reliable performance of SRCSM sensors. **a** Real-time relative resistance variation ratios (Δ*R*/*R*_0_) of the SRCSM sensor under stretching strain. **b** Real-time relative capacitance variation ratios (Δ*C*/*C*_0_) of the SRCSM sensor under pressure. The insets: The response and recovery times. **c** Durability test of the SRCSM sensor under 30% strain during 5000 cycles with a stretching loading rate of 200.1 mm/min. **d** Durability test of the SRCSM sensor under 14 kPa pressure for 5000 cycles with a pressure loading rate of 0.5 mm/min. **e** Dynamic responses of the SRCSM sensor with frequency changes from 0.05 to 0.13 Hz under 30% strain, **f** Dynamic responses of the SRCSM sensor with the changed frequency from 0.2 to 1.0 Hz under 14 kPa

To investigate the real-time reliability of the SRCSM sensor, the relative resistance and capacitance variation ratios (Δ*R*/*R*_0_, Δ*C*/*C*_0_) at stretching strains of 30%, 60%, 90%, and 120% and pressures of 2 kPa, 4 kPa, and 14 kPa under four loading–unloading cycles are depicted in Fig. 5. Each loading and unloading stimulus lasted for 20 s to investigate the stability of the sensor. It can be observed that Δ*R*/*R*_0_ and Δ*C*/*C*_0_ gradually increased with increasing loaded stimuli (the stretching strains rose from 30% to 120%, and the pressures increased from 2 kPa to 14 kPa). Moreover, Δ*R*/*R*_0_ and Δ*C*/*C*_0_ were maintained at stable values under each applied stimulus. These results indicate that the SRCSM sensor can monitor Δ*R*/*R*_0_ and Δ*C*/*C*_0_ under stimuli simultaneously. The insets in Fig. 5a, b illustrate that the response and recovery times are approximately 300 ms under a stretching strain of 30% and a pressure of 14 kPa, indicating a fast stimulus response, which can be satisfied to monitor human motions^26–30^.

Stability and durability are essential for applications of wearable sensors. Figure 5c, d shows the Δ*R*/*R*_0_ of the SRCSM sensor under 30% strain and Δ*C*/*C*_0_ of the SRCSM sensor under 14 kPa pressure during 5000 cycles. Δ*R*/*R*_0_ and Δ*C*/*C*_0_ were stable with good repeatability during the whole durability test. The inset shows the stable signal output during the testing process. Furthermore, due to the highly tough overall structure, the utilized sensor also offers good dynamic performance in reproducibility and stability under different loaded stimuli frequencies, as shown in Fig. 5e, f. More details about the Δ*R*/*R*_0_ performance under pressure and Δ*C*/*C*_0_ performance under strain are shown in Fig. S3.

Table 1 compares the essential aspects of the multimode sensors reported in recent literature with our work. From the comparison, it can be declared that most single devices cannot collect and decouple multiple parameters simultaneously. Additionally, the assembly of two devices increases the complexity of the fabrication process. In contrast, our SRCSM sensor exhibits excellent decoupling capability under the premise of convenient manufacture and low cost. More comparison information is listed in Table S2, which indicates that the presented SRCSM sensor has excellent overall performance.

**Table 1:** Summary of the recent multimode sensors compared with the proposed SRCSM sensor

Multimode	Fabrication^a^	Structure	Conformality^b^	Costs	Decoupling	Ref.
Stretching strain, Twist	Simple	Single device	Middle	Low	No	^13^
Multidirectional shearing	Simple	Single device	Poor	Low	No	^14^
Pressure, Shearing, Bending	Complex	Single device	Middle	High	No	^15^
Stretching strain, Pressure	Simple	Single device	Middle	Low	No	^16^
Pressure, Shearing	Complex	Single device	Poor	High	No	^48^
Stretching strain, Pressure	Complex	Two devices assembled	Middle	High	Yes	^19^
Stretching strain, Pressure	Complex	Two devices assembled	Good	High	Yes	^20^
Stretching strain, Bending	Complex	Two devices assembled	Poor	Low	Yes	^21^
Multidirectional stretching strain	Complex	Two devices assembled	Middle	High	Yes	^22^
Stretching strain, Pressure, shearing	Complex	One device integrated two structures	Poor	Low	Yes	^23^
Stretching strain, Pressure	Complex	One device integrated two structures	Poor	High	Yes	^24^
Stretching strain, Pressure	Simple	One device integrated two structures	Middle	Low	Yes	This work

^a^The judgment of fabricating complexity in the table is mainly obtained by measuring and comparing the number of steps in the preparation process and the difficulties of the process.

^b^The definition and evaluation of ‘conformality’ shown in Table 1 are mainly considered from the thickness of the device. Conformality decreases as the thickness of the sensor increases. (The specific device size is described in detail in Table S2.)

### Application in Human Motion Monitoring and Recognition

**Fig. 6:**
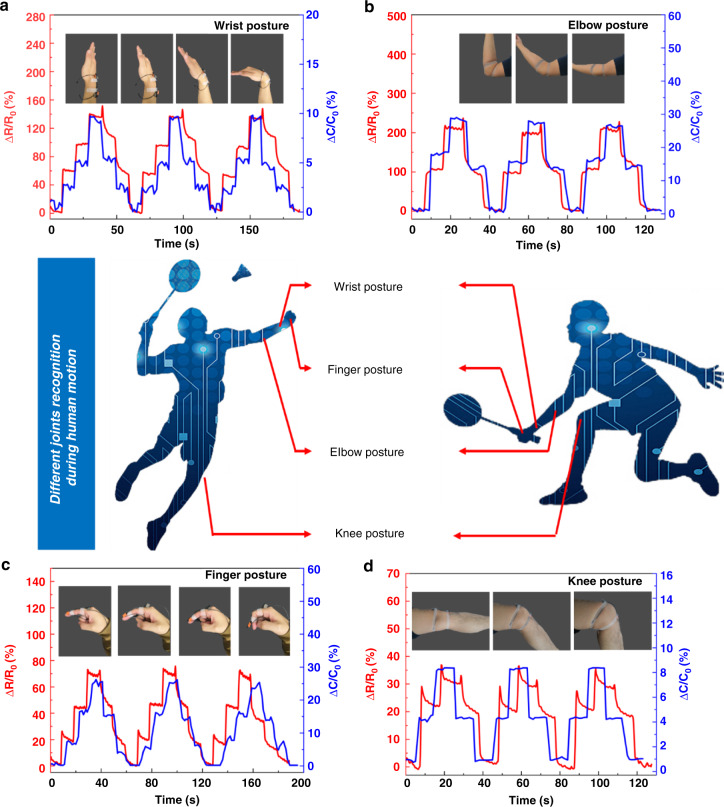
Demonstrations of potential applications of the SRCSM sensor over a wide pressure and strain range. **a** Response of the sensor under 30°, 60°, and 90° bending of the wrist. **b** Response of the sensor under 45° and 90° bending of the elbow. **c** Response of the sensor under 30°, 60°, and 90° bending of the finger. **d** Response of the sensor under 45° and 90° bending of the knee

To demonstrate the great potential applications of the proposed SRCSM sensor in the wearable electronic field, the real-time physical signals of the joint bending (finger, elbow, wrist, and knee) caused by human motions are measured. As shown in Fig. 6, the states of different joint activities are monitored by two sensor modes, and each action is distinguished by both Δ*C*/*C*_0_ and Δ*R*/*R*_0_ simultaneously. Figure 4 shows that the Δ*R*/*R*_0_ of the SRCSM sensor mainly depends on the stretching strain, while Δ*C*/*C*_0_ depends on the pressure. For the elbow joint, due to the protrusion of the joint bone and the large skin surface stretch, ΔR/R0 and Δ*C*/*C*_0_ are very large, which belongs to the High Δ*R*/*R*_0_-High Δ*C*/*C*_0_ state. For the finger joint, the protrusion of the bone causes the sensor to be compressed when the finger joint is bent, resulting in a relatively large Δ*C*/*C*_0_. The small area of the stretching strain makes Δ*R*/*R*_0_ smaller than that of the elbow joint and wrist joint. Thus, finger joint bending corresponds to a low Δ*R*/*R*_0_- high Δ*C*/*C*_0_ state. Since there is no protruding bone, the Δ*C*/*C*_0_ of the wrist joint is mainly affected by stretching strain, which is relatively small. The stretching strain of skin also leads to a significant Δ*R*/*R*_0_. It can be classified as a High Δ*R*/*R*_0_-Low Δ*C*/*C*_0_ state. For the knee joint, due to the apparent curvature of the kneecap even when laid flat, the conformally adhered sensor on the kneecap cannot detect enough strain, and Δ*R*/*R*_0_ and Δ*C*/*C*_0_ are both small during the motion of the knee joint, which is consistent with Low Δ*R*/*R*_0_-Low Δ*C*/*C*_0_ state corresponds.

**Fig. 7:**
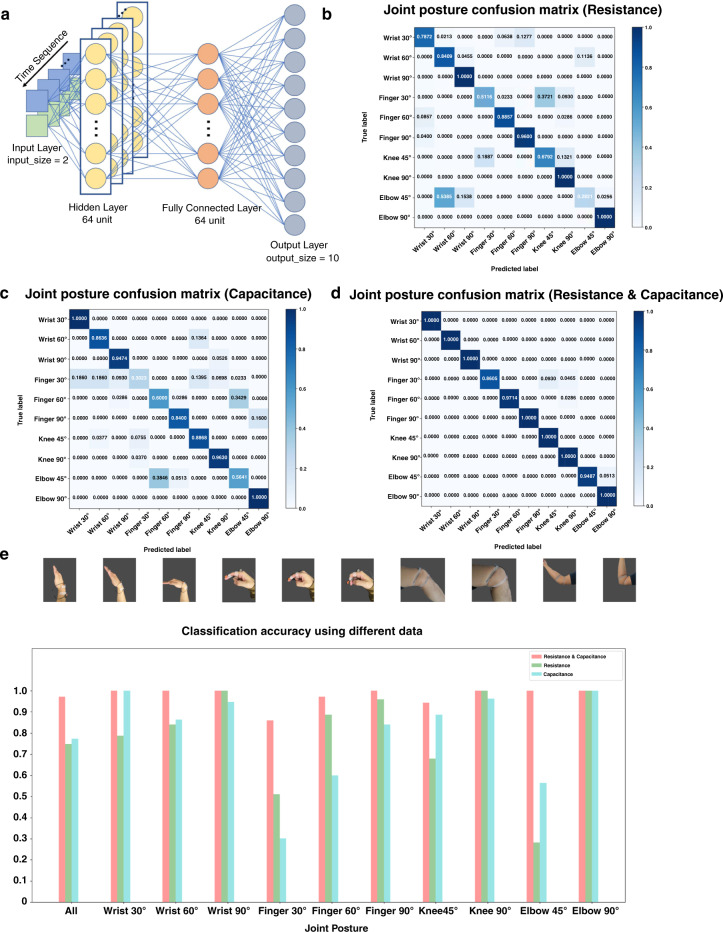
Application in different postures recognition. **a** Schematic illustration of the long short-term memory (LSTM) deep-learning algorithm. **b**–**d** Classification confusion matrices using only resistance data, capacitance data, and both resistance and capacitance data. **e** Histogram of classification accuracy of all joint postures using different data

Furthermore, due to the decoupled dual signal output characteristics of the SRCSM sensor, we applied the long short-term memory (LSTM) deep-learning algorithm to classify the electronic signals of different postures with different joints for recognition training. The LSTM neural network introduces the mechanism of the gating units based on the recurrent neural network (RNN)^31–33^. The gating mechanism controls the forgetting, input, and output of information, solving long-term dependence^34–36^. Figure 7a shows a schematic illustration of the artificial neural network algorithms. The input size of the LSTM layer is 2, corresponding to the input resistance and capacitance data, the number of hidden layers is 1, and the output size is 10, corresponding to 10 categories (wrist 30°, 60°, 90°; finger 30°, 50°, 90°; knee 45°, 90°, and elbow 45°, 90°). The softmax layer receives the output of the fully connected layer and completes the classification of the samples. The training set and the test set were divided at a ratio of 4:1, and the classification performance was evaluated based on the classification accuracy. Figure 7b–d shows the classification confusion matrix results using only resistance data, only capacitance data, and both resistance and capacitance data, respectively. In the case of using only electrical resistance data, the LSTM classifier does not perform well for the classification of wrist joints 30°, wrist joints 60°, finger joints 30°, knee joints 45°, and elbow joints 45°, and the classification accuracy is less than 85%. When only the capacitance data are used, the classifier has a poor classification effect for finger joints 30°, finger joints 60°, and elbow joints 45°. A large number of elbow joint samples of 45° are misclassified as knee 60°. It is speculated that the compressing degree of the elbow joint 45° sensor is similar to that of the finger joints 60°, and the classification accuracy is less than 56.4%. The results show that it is challenging to distinguish postures using a single capacitance or resistance signal effectively. When using both resistance and capacitance data, the classification accuracy of finger joint 30°, knee joint 45°, and elbow joint 45° is 94.64%, 97.78%, and 94.44%, respectively. Moreover, the classification accuracies of other joint postures reach 100%, which is significantly improved. Figure 7e displays the classification accuracy for all joint poses, and the highest classification accuracy is achieved by using both datasets. The overall classification accuracy rates of using only resistance data, capacitance data, and both resistance and capacitance data are 74.79%, 77.36%, and 97.13%, respectively, which indicates that the SRCSM sensor has the highest classification accuracy in distinguishing postures.

Through the above analysis, the joint types and the bending degree of joints during motions can be distinguished by two independent output signals simultaneously from the SRCSM sensor. It has a meaningful advantage in scientific sports training, innovative medicine, and other fields. For example, while playing badminton, the signal of different joints can be used to recognize different postures, such as smashing, cutting, driving, and so on. Different sports, such as playing badminton and cycling, make the corresponding changes in different joints entirely different.

Due to the outstanding sensing performance, the proposed sensor not only detects large deformations but also monitors subtle motions such as voice recognition and pulse signals. The value of the relative resistance changes for different words and sentences is comparable with the existing research^37–39^. Additionally, the voice recognition system exhibits almost identical voltage waveforms and frequency responses during the repeatability tests, demonstrating high reproducibility and reliability. For pulse motion, characteristic peaks of the three human sphygmic waveforms^40–43^ relevant to percussion waves (P-waves), tidal waves (T-waves), and diastolic waves (D-waves) can be distinguished. The peak of relative resistance changes is nearly 0.5%, which shows superior performance compared with other studies^44–47^. Detailed information is shown in Fig. S4, demonstrating that the SRCSM sensor has outstanding performance in real-time monitoring of subtle motion areas.

## Conclusion

In summary, we demonstrated a wearable integrated SRCSM sensor based on a low-cost and convenient layer-by-layer casting fabrication process. This multimode sensor was integrated into a unique seamless structure, which simultaneously consisted of two main parts to decouple the different stimuli by an independent resistance-capacitance sensing mechanism. Benefitting from this excellent decoupling capability, the sensor can differentiate the motion characteristics of the positions and states of different joints with precise recognition (97.13%) with the assistance of deep learning algorithms. In addition, subtle signals such as vocalization and pulse beats can also be detected due to the high sensitivity of the resistive component. Although this study is only a proof of concept demonstration, we anticipate that this simple, lost-cost but efficient strategy could be a prospective candidate for wearable applications in portable electronic skin, health care and intelligent sports monitoring devices, advanced human–machine interfaces, and intelligent soft robot perception systems.

## Experimental section

### Materials

MWCNTs with an average diameter of 50 nm and a length less than 10 μm were purchased from XFNANO Inc. (Nanjing, China). Ecoflex was obtained from Smooth-On, Inc. (Macungie, U.S). The conductive metal tape was brought from MaoYe Inc. (Shenzhen, China). All the materials were utilized as received without any further purification.

### Characterization

The morphology of the SRCSM sensor was observed via SEM (Zeiss Ultra Plus) at an acceleration voltage of 5 kV. The mechanical measurements were accomplished by a Mark-10 machinal testing machine (ESM303 force test stands and SERICE 5 digital force gauges). Electrical measurements were tested by a setup test system, including an LCR digital bridge (TH2826, TONGHUI) and a semiconductor parameter analyzer (Keithley 4200-SCS).

## Supplementary information

Supplementary Information
