# Supplementary material for: Wearable multimode sensor with a seamless integrated structure for recognition of different joint motion states with the assistance of a deep learning algorithm
Source: Microsyst Nanoeng. 2022 Feb 17;8:24. doi: 10.1038/s41378-022-00358-2 (PMC8854609; doi:10.1038/s41378-022-00358-2)
Supplement: Supplementary file 1 — Supplementary Information [file 41378_2022_358_MOESM1_ESM.docx]

**Supplementary information**

**Wearable Multi-mode Sensor with Seamless Integrated Structure for Different Joints Motion States Recognition with the Assistance of Deep Learning Algorithm**

Lei Wen, Meng Nie*, Pengfan Chen, Yu-na Zhao, Jingcheng Shen, Chongqing Wang, Yuwei Xiong, Kuibo Yin, Litao Sun*

SEU-FEI Nano-Pico Center, Key Laboratory of MEMS of Ministry of Education, School of Electronic Science & Engineering, Southeast University, Nanjing, 210096, P. R. China.


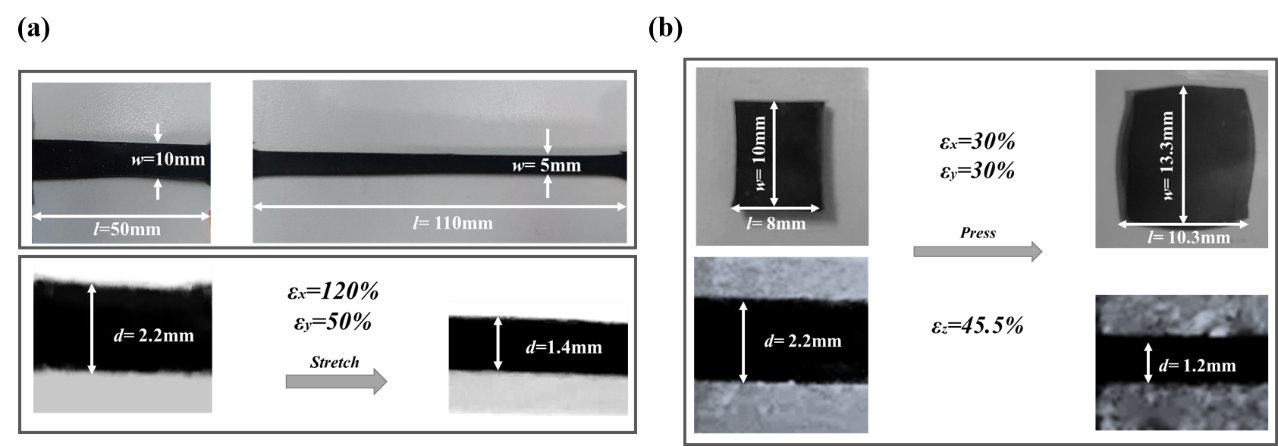


Fig. S1. (a) The Optical image of sensor's dimensional change under tensile strain. (b) Theoretical derived values of relative capacitance changes (Δ*C/C*_0_) as a function of applied pressure (c) The Optical image of sensor's dimensional change under pressure. (d) Theoretical derived values of relative resistance changes (Δ*R/R*_0_) as a function of applied pressure

**Part. 1：Detailed formula derivation of Δ*C/C*_0_ under stretching strain**

The formula of the variation ratio of capacitance under stretching strain is derived.

According to the definition of Poisson's ratio, Δ*w* and Δ*d* can be expressed by Δ*l* as follows

$\triangle w=-w_{0}\frac{\triangle l}{l_{0}}*\nu_{xy}$ （S1）

$\triangle d=-d_{0}\frac{\triangle l}{l_{0}}*\nu_{xz}$ （S2）

where $\nu$_xy_ and $\nu$_xz_ are Poisson's ratios in the *y* and *z* directions, respectively. Substitute Equation S1 and S2 into Equation 2, the capacitance *C* can be defined as

$C=\frac{\varepsilon_{r}w_{0}l_{0}\left( 1-\frac{\triangle l}{l_{0}}*\nu_{xy} \right)\left( 1+\frac{\triangle l}{l_{0}} \right)}{d_{0}(1-\frac{\triangle l}{l_{0}}*\nu_{xz})}$ （S3）

the change of the capacitance Δ*C* and the variation ratio of capacitance Δ*C/C*_0_ can be achieved.

$\triangle C= \frac{\varepsilon_{r}w_{0}l_{0}}{d_{0}}\left[ \frac{\left( 1-\frac{\triangle l}{l_{0}}*\nu_{xy} \right)\left( 1+\frac{\triangle l}{l_{0}} \right)}{1-\frac{\triangle l}{l_{0}}*\nu_{xz}}-1 \right]$ （S4）

$\frac{\triangle C}{C_{0}}=\frac{\left( 1-\frac{\triangle l}{l_{0}}*\nu_{xy} \right)\left( 1+\frac{\triangle l}{l_{0}} \right)}{1-\frac{\triangle l}{l_{0}}*\nu_{xz}}-1$ （S5）

**Part.2：Detailed formula derivation of Δ*C/C*_0_ under pressure**

The formula of the variation ratio of capacitance under pressure is also derived.

*ν*_zx_ and *ν*_zy_ are Poisson's ratios in the *x* and *y* directions, respectively.*Δw* and *Δl* can be expressed as follows

$\triangle w=-w_{0}\frac{\triangle d}{d_{0}}*\nu_{zx}$ （S6）

$\triangle l=-l_{0}\frac{\triangle d}{d_{0}}*\nu_{zy}$ （S7）

Substitute Equation S6 and S7 into Equation 2, the capacitance *C* can be derived as

$C=\frac{\varepsilon_{r}w_{0}l_{0}\left( 1+\frac{\triangle d}{d_{0}}*\nu_{zx} \right)\left( 1+\frac{\triangle d}{d_{0}}*\nu_{zy} \right)}{d_{0}(1-\frac{\triangle d}{d_{0}})}$ （S8）

the change of the capacitance *ΔC* and the variation ratio of capacitance *ΔC/C_0_* can also be achieved as

$\triangle C= \frac{\varepsilon_{r}w_{0}l_{0}}{d_{0}}\left[ \frac{\left( 1+\frac{\triangle d}{d_{0}}*\nu_{zx} \right)\left( 1+\frac{\triangle d}{d_{0}}*\nu_{zy} \right)}{1-\frac{\triangle d}{d_{0}}}-1 \right]$ （S9）

$\frac{\triangle C}{C_{0}}=\frac{\left( 1+\frac{\triangle d}{d_{0}}*\nu_{zx} \right)\left( 1+\frac{\triangle d}{d_{0}}*\nu_{zy} \right)}{1-\frac{\triangle d}{d_{0}}}-1$ （S10）


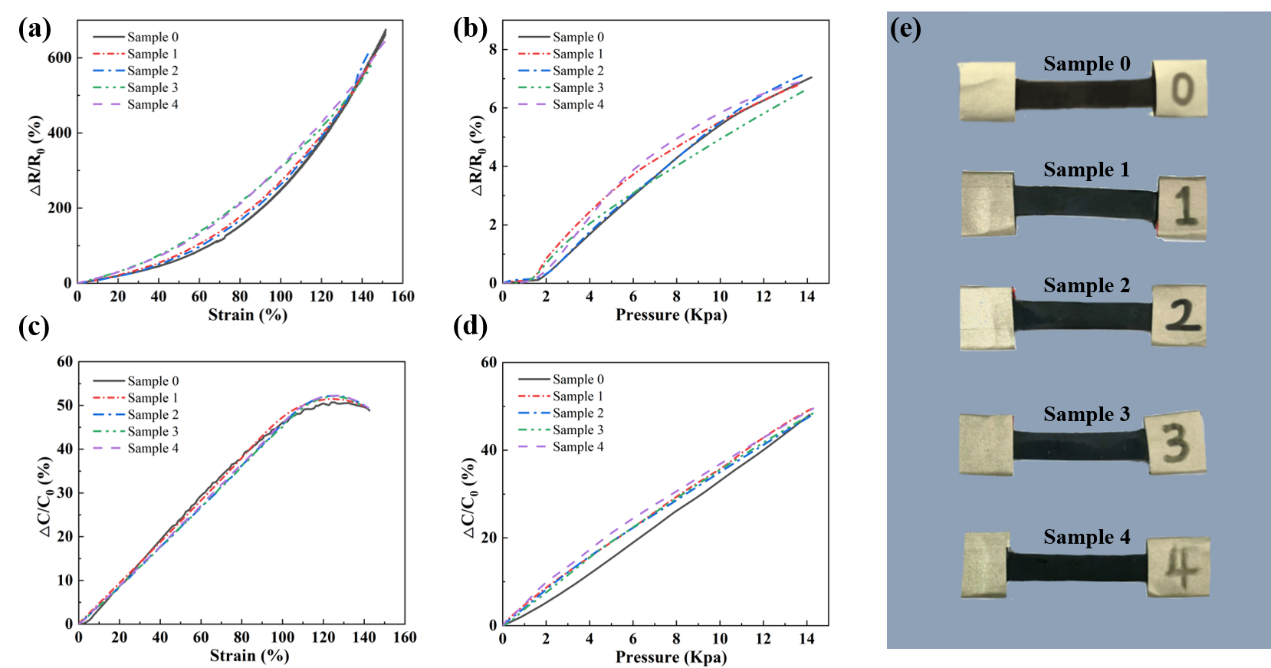
 Fig. S2. (a)-(d) The sensitivity curves of different batches of samples under different modes (e) The photograph of different batches of samples.

Tab S1. The sensitivity of different batches of samples under different modes

| **Sample**  **Sensitivity** | **Sample 0** | **Sample 1** | **Sample 2** | **Sample 3** | **Sample 4** |
| --- | --- | --- | --- | --- | --- |
| **Resistance-Strain (GF)** | 2.0  (0-80%)  7.6  (80-140%) | 2.3  (0-80%)  7.2  (80-140%) | 2.2  (0-80%)  7.3  (80-140%) | 2.4  (0-80%)  7.4  (80-140%) | 2.4  (0-80%)  7.1  (80-140%) |
| **Resistance-Pressure (S)**  **（kPa^-1^）** | 0.57 | 0.54 | 0.60 | 0.54 | 0.59 |
| **Capacitance-Strain (GF)** | 0.49 | 0.48 | 0.46 | 0.47 | 0.46 |
| **Capacitance-Pressure (S)**  **（kPa^-1^）** | 3.4 | 3.4 | 3.4 | 3.5 | 3.5 |

The experimental results of the performance in Fig. S2 and Tab S1 illustrated that the performance of different batches of samples is stable. The GF value of the resistance-strain is 2.0~2.4 (0-80% strain) and 7.2~7.6 (80%-140% strain). The GF value of capacitance-strain is 0.46~0.49 (0-140% strain). The S value of the resistance-pressure and capacitance-pressure are from 0.54~0.60 kPa^-1^ and 3.4~3.5 kPa ^-1^ (0-14 kPa pressure), respectively. (The fluctuation is caused by the measurement error). In addition, the amount of MWCNTs was 1mg/cm^2^. It can be seen from the SEM images in Fig. 2f-h that the brushed MWCNTs are evenly distributed on the Ecoflex surface. Moreover, the initial resistance of the resistive component of the five samples are 22.0 kΩ, 21.8 kΩ, 23.2 kΩ, 23.8 kΩ, 21.9 kΩ, respectively, the average initial resistance is 22.54 kΩ. The morphology characterization and the electrical properties of the different samples illustrate the random MWCNTs have been evenly dispersed on the Ecoflex surface to obtain the stable performance of different batches of devices.


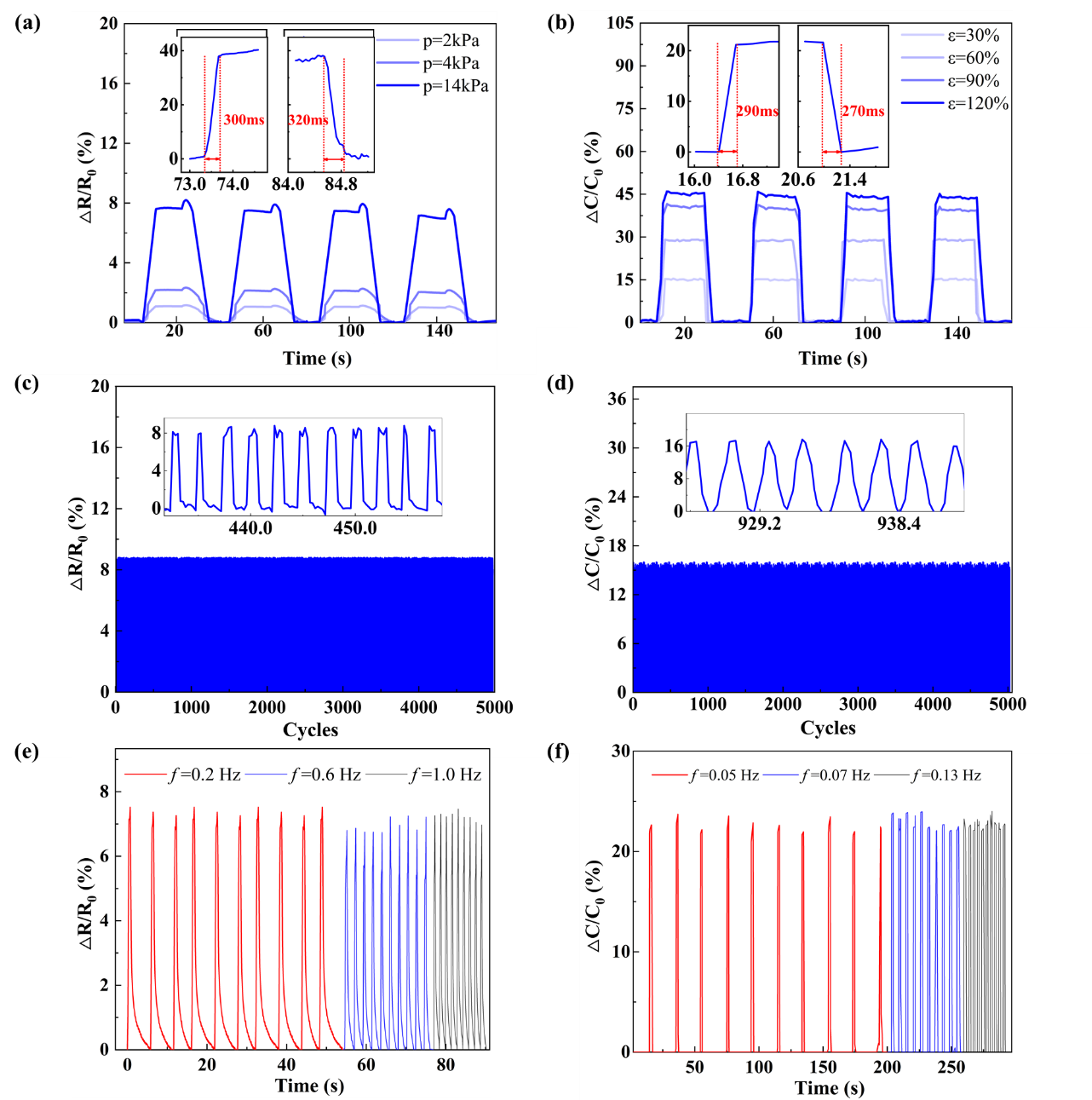


Fig. S3. (a) Real-time relative resistance variation ratios (Δ*R*/*R*_0_) of the SRCSM sensor under the pressure. (b) Real-time relative capacitance variation ratios (Δ*C*/*C*_0_) of the SRCSM sensor under the stretching strain. The insets: The response and recovery times. (c) The durability test of the SRCSM sensor under 14 kPa pressure during 5000 cycles with the stretching loaded rate of 200.1 mm/min. (d) The durability test of the SRCSM sensor under 30% strain during 5000 cycles with the pressure loaded rate of 0.5 mm/min. (e) Dynamic responses of the SRCSM sensor with the changed frequency from 0.2 Hz to 1.0 Hz under 14 kPa, (f) Dynamic responses of the SRCSM sensor with the changed frequency from 0.05 Hz to 0.13 Hz under 30% strain.

Tab S2. Summary of parameters and performance of the recent multi-mode sensors compared with the proposed SRCSM sensor.

| Materials | Sizes(mm) | Range | Sensitivity | Mechanical properties | Cycles | Ref. |
| --- | --- | --- | --- | --- | --- | --- |
| PI，Ag，PDMS | 20*8.52*1.10 | 60% strain  400° twist | GF=4000 | N/A | 2000 | [13] |
| PDMS, MCCA | 5*15*5 | 10% stretch | 45°: GF=0.5  Horizontal: GF= 1.1  Perpendicular: GF=0.2 | N/A | 50 | [14] |
| PDMS，PI，Cr/Au，Si，Ti/Sio2 | 2*2*1 | 50kPa | Pressure: S=0.001kPa^-1^  Shearing: S=0.007N^-1^ | N/A | 1000 | [15] |
| CNTs/HAPAAm Hydrogel | 10*10*2 | 1000% strain  50kPa pressure | GF=4.32  S=0.127 kPa-1 | Youngs Modulus: 267kPa  Fracture strain:3000% | Stretch:200  Press:300 | [16] |
| CNT，PDMS conductive silicone | 15*15*5 | 128 Pa ~ 44 kPa pressure  5.28 kPa ~ 12.9 kPa shearing | Pressure：S=0.0173kPa^-1^ Shearing：S=0.165kPa^-1^ | N/A | N/A | [31] |
| PGS, POMaC | 22*10*1.65 | 15% strain  10kPa pressure | GF=3.3  S:0.7 kPa^-1^(0-1kPa)  0.13 kPa^-1^(5-10kPa) | Youngs Modulus: 500kPa | Stretch:20000  Press:30000 | [19] |
| CNT, PDMS | 5*20*0.4  5*20*0.2 | 50% stretch | HA-CNT-PDMS  GF=594(15%)  GF=65(50%)  VA-CNT-PDMS  GF=326 (25%)  GF=52 (50%) | HA：5000kpa；2000kpa  VA：8000kpa；2400kpa | 6000 | [20] |
| Nacl/AG，2-CEA/PEGDA | 100*5*5 | 500% stretch  0-90° bending | GF=12 | N/A | N/A | [21] |
| PAN，CNF，EGaIn，PDMS | 25*5*1 | 30% stretch | Lateral：GF=180  Longitudinal：GF=0.3 | Youngs Modulus: 953kPa | 2500 | [22] |
| PDMS， AgNWs, CNFs | 60*20*6 | 50% stretch  50% compressive  30% shearing | stretch：2.29/8.21/0.81  compressive：0.63/1.58/-2.14  shearing：0.37/1.41/0.004 | Youngs Modulus: 100kPa  Fracture strain:55% | 5000 | [23] |
| PDMS/SWNT-film/porous PDMS | 10*10*4 | 30% stretch  25kPa pressure | Capacitance:  Strain: GF=1.3 Pressure:  S=0.7kPa^-1^(0-1kPa)  S=0.14kPa^-1^(1-5kPa)  S=0.005kPa^-1^ (>5kpa)  Resistance:  Strain: GF=0.65 Pressure: S=0 | N/A | 1000 | [24] |
| CNT，Ecoflex | 50*10*2.3 | 140% stretch  14kPa pressure | Capacitance: Strain: GF=0.49 Pressure:  S=3.4kPa^-1^  Resistance:  Strain:  GF=2.02(0-80%)  GF=7.62 (80%-150%)  Pressure:  S=0.15kPa^-1^(0-2kPa)  S=0.57 kPa^-1^ (2-14kPa) | Youngs Modulus: 269kPa  Fracture strain:458% | 5000 | This work |


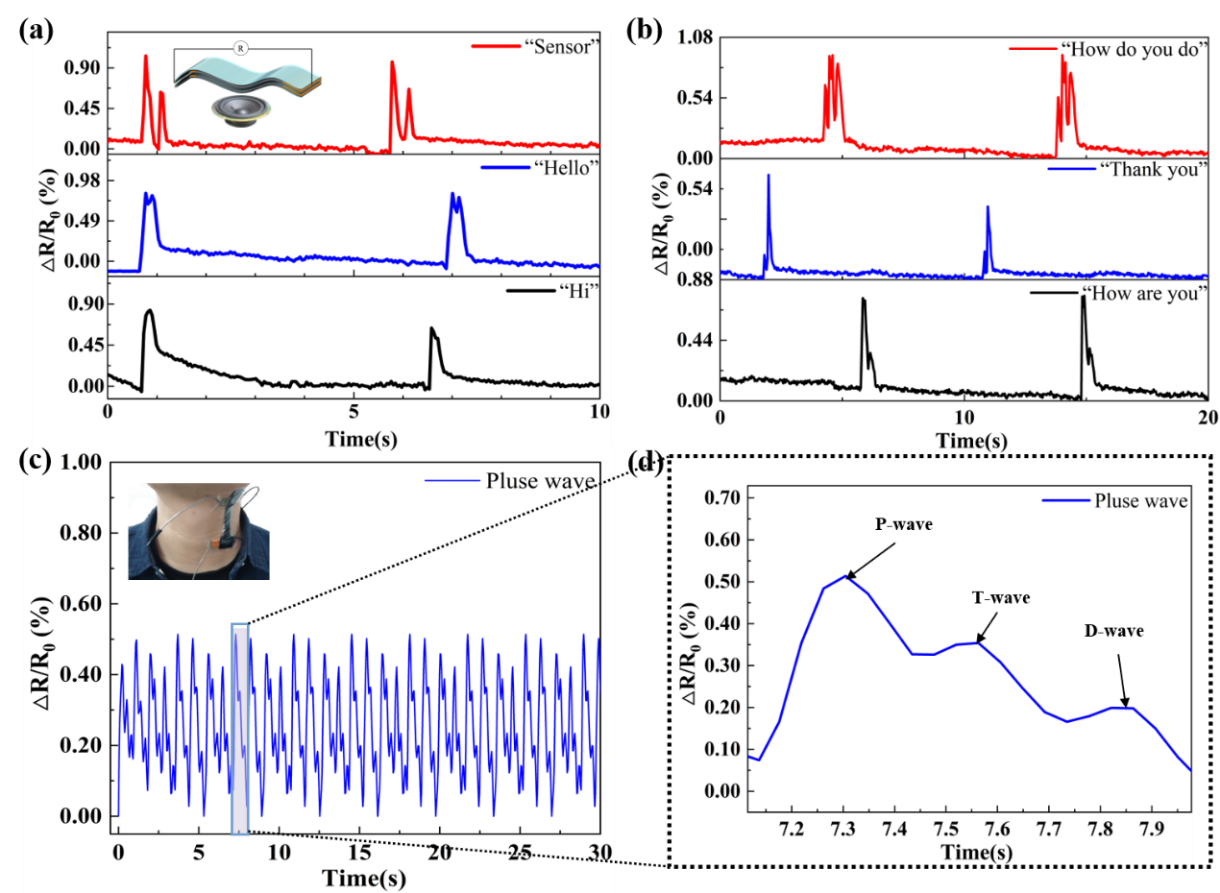


Fig. S4. The demonstrations of potential applications in the low-pressure range. (a) The resistance response of the sensor for voice recognition, including different words, (b) The resistance response of the sensor for voice recognition, including different sentences, (c)-(d) The resistance response of the SRCSM sensor on the neck to monitor the pulse wave.

As the schematic illustration is shown in Fig. S4a-b, a simple voice recognition system is set up, including the speaker, the SRCSM sensor, and the semiconductor parameter analyzer. The output signal for the American pronunciations of “Hi”, “Hello” and” Sensor” are displayed in Fig. S4. The resistance variation ratios Δ*R*/*R*_0_ of the sensor exhibit different waveforms and distinguishing feature points because the speaker produces different vibration waves when different words are pronounced. By comparing the number and amplitude of peaks, these words can be successfully distinguished. To further prove the feasibility of the application, sentences such as “How are you”, “Thank you”, and “How do you do” are also used for voice recognition tests. As illustrated in Fig. S4(b), all of them can be accurately identified.

The sensor can also be applied as a noninvasive pulse monitoring system to collect human-being pulse waveforms. Fig. S4(c) displays the real-time pulse signals recorded by the proposed sensor. Characteristically peaks of the three human sphygmic waveforms relevant to percussion wave (P-wave), tidal wave (T-wave), and diastolic wave (D-wave) can be distinguished in Fig. S4(d). The P-wave is the early systolic peak pressure, T-wave is the late systolic peak pressure, and D-wave appears in the diastole region and is referred to as the diastolic pulse waveform.
